# Analysis between ABO blood group and clinical outcomes in COVID-19 patients and the potential mediating role of ACE2

**DOI:** 10.3389/fmed.2023.1167452

**Published:** 2023-06-23

**Authors:** Xianfei Zeng, Hongyan Fan, Jinxin Kou, Dongxue Lu, Fang Huang, Xi Meng, Haiying Liu, Zhuo Li, Mei Tang, Jing Zhang, Nannan Liu, Xingbin Hu

**Affiliations:** ^1^School of Medicine, Northwest University, Xi'an, China; ^2^Xi'an Area Medical Laboratory Center, Xi’an, China; ^3^Department of Blood Transfusion, 940 Hospital, Lanzhou, China; ^4^Department of Anesthesiology and Perioperative Medicine, Xijing Hospital, Xi’an, China; ^5^Department of Laboratory Medicine, Xi’an Chest Hospital, Xi’an, China; ^6^Department of Laboratory Medicine, First Affiliated Hospital of Xi'an Medical University, Xi’an, China; ^7^Department of Blood Transfusion, Tangdu Hospital, Xi’an, China; ^8^Department of Blood Transfusion, Xijing Hospital, Xi’an, China; ^9^Intensive Care Center, Xijing Hospital, Xi’an, China

**Keywords:** COVID-19, ABO blood type, susceptibility, ARDS, AKI, ACE2

## Abstract

Severe acute respiratory syndrome coronavirus 2 (SARS-CoV-2) has become the most common coronavirus that causes large-scale infections worldwide. Currently, several studies have shown that the ABO blood group is associated with coronavirus disease 2019 (COVID-19) infection and some studies have also suggested that the infection of COVID-19 may be closely related to the interaction between angiotensin-converting enzyme 2 (ACE2) and blood group antigens. However, the relationship between blood type to clinical outcome in critically ill patients and the mechanism of action is still unclear. The current study aimed to examine the correlation between blood type distribution and SARS-CoV-2 infection, progression, and prognosis in patients with COVID-19 and the potential mediating role of ACE2. With 234 patients from 5 medical centers and two established cohorts, 137 for the mild cohort and 97 for the critically ill cohort, we found that the blood type A population was more sensitive to SARS-CoV-2, while the blood type distribution was not relevant to acute respiratory distress syndrome (ARDS), acute kidney injury (AKI), and mortality in COVID-19 patients. Further study showed that the serum ACE2 protein level of healthy people with type A was significantly higher than that of other blood groups, and type O was the lowest. The experimental results of spike protein binding to red blood cells also showed that the binding rate of people with type A was the highest, and that of people with type O was the lowest. Our finding indicated that blood type A may be the biological marker for susceptibility to SARS-CoV-2 infection and may be associated with potential mediating of ACE2, but irrelevant to the clinical outcomes including ARDS, AKI, and death. These findings can provide new ideas for clinical diagnosis, treatment, and prevention of COVID-19.

## Introduction

Severe acute respiratory syndrome coronavirus 2 (SARS-CoV-2) has become the third coronavirus that causes large-scale infections among humans, following severe acute respiratory syndrome coronavirus (SARS-CoV) and Middle East respiratory syndrome coronavirus in the past two decades (1, 2). The severe outbreak of coronavirus disease 2019 (COVID-19) made it a public health threat all over the world (3).

It is meaningful to perform relevant research on the susceptibility and outcome of COVID-19. Blood group antigens are genetically predisposed to various diseases such as diabetes and breast cancer, closely related to acute respiratory distress syndrome (ARDS) and acute kidney injury (AKI), and may also be involved in the infection process of various viruses such as SARS-CoV and norovirus (4–8). Studies have shown that type A antigen has a significant susceptibility to SARS-CoV-2 (9–11), and it is the third biological marker of susceptibility that has received wide attention after men and the elderly as susceptibility factors (12, 13).

The mechanism of blood group-related SARS-CoV-2 infection may be closely related to the interaction between blood group antigen and angiotensin-converting enzyme 2 (ACE2). The main evidence includes: first, the receptor binding domain (RBD) of S protein binds to ACE2 epithelial cells and thus enters into the cell, which is the main mechanism of SARS-CoV-2 infection (14). ACE2 is considered to be a key receptor for SARS-CoV-2 to invade human cells (15). In the first SARS-CoV-2 transgenic mouse model established, the expression level of ACE2 was found to be positively correlated with the degree of novel coronavirus infection within a certain range (16). ACE2 is found not only in the lungs, but also in the testes, heart, kidneys, and tissues lining blood vessels (17–20). Second, viral apices (VP8) play an important role in rotavirus invasion of enteroepithelial cells, and spike proteins (S proteins) similar to SARS-CoV-2 infect respiratory epithelial cells (21). Rotavirus studies have shown that the A group antigen is the only glycan that interacts with VP8, thus leading to viral susceptibility to the A antigen (21, 22). According to the current evidence, the difference in blood group susceptibility of SARS-CoV-2 is most likely related to the binding or invasion of the S protein (21–24).

This multicenter study analyzed the relationship between blood group antigen and the onset, progression, and prognosis of COVID-19 in the Chinese population. In terms of mechanism study, we proposed the hypothesis that blood group antigens could be biomarkers of SARS-CoV-2 susceptibility by influencing the interaction between ACE2 and S proteins. This study explored the infection mechanism and invasion mechanism of SARS-CoV-2 related to the ABO blood group at the protein and gene levels, thus providing A complete chain of evidence for the susceptibility of type A to severe disease.

The aim of this study was to elucidate the possibility of ABO blood group antigen as a biological marker of SARS-CoV-2 susceptibility and the related mechanism. In this study, the susceptibility of blood group antigen to SARS-CoV-2 and the differences in the transformation of patients with different blood groups to severe disease were verified epidemiologically, and the biological mechanism of blood group susceptibility was partially elucidated through protein-cell interaction.

## Materials and methods

### Study design

#### Study populations

Two retrospective cohorts diagnosed with COVID-19 were enrolled in this study between February 5 and March 20, 2020. The first cohort comprised patients with mild symptoms admitted to three hospitals in Xi’an, Beijing, and Wuhan. The second cohort comprised critically ill patients from the two hospitals in Xi’an and Wuhan. Five hospitals in this study were assigned by the government as treatment centers for COVID-19 patients. All study participants were of Chinese Han nationality population and aged >13 years. In the critical cohort, patients were excluded if they died within 24 h upon admission or had current evidence of congestive heart failure, myocardial infarction, or severe isolated head trauma, which was not related to the progress of COVID-19. Study have suggested that the infection of COVID-19 may be related to the interaction between ACE2 and blood group antigens (14, 15). Therefore, we performed the evaluation of ACE-2 protein levels in healthy subjects to verify our hypothesis. The blood samples were taken from healthy participants at a hospital in Xi’an, China. Demographic data of the healthy participants were shown in Table 1. Informed consent was obtained from all patients or first-grade relatives.

**Table 1:** Demographic data of healthy participants.

Characteristics	A (*n* = 20)	B (*n* = 20)	O (*n* = 20)	AB (*n* = 19)	*P-*value	All participants (*n* = 79)
Age (years)	34 (26, 45)	37 (24, 42)	33 (27, 51)	35 (29,47)	0.579	34.5 (24, 49)
Male	13 (65.0%)	15 (75.0%)	12 (60.0%)	11 (57.9)	0.681	51 (64.6%)
Female	7 (35.0%)	5 (25.0%)	8 (40.0%)	8 (42.1)		28 (35.4%)

Data are shown as *n* (%) for categorical variables and median (interquartile range) for continuous variables.

### Diagnosis and outcome definitions

COVID-19 was defined according to the World Health Organization interim guidance (25). COVID-19 was confirmed by a positive real-time reverse transcriptase polymerase-chain-reaction test of SARS-CoV-2 on nasal and pharyngeal swab specimens from suspected patients. Critically ill patients were defined as those admitted to the intensive care unit (ICU) who required mechanical ventilation or had a fraction of inspired oxygen (FiO_2_) of ≥60% (26, 27). Identification of critically ill patients was achieved by reviewing and analyzing the admission logs and histories from all available electronic medical records. AKI was diagnosed if the serum creatinine levels change within 2 days based on the Acute Kidney Injury Network (AKIN) criteria (28). Patients were diagnosed with ARDS based on the 2012 Berlin definition (29).

### Laboratory testing and data collection

ABO blood type of patients was determined by standard red blood cell (RBC) typing performed for clinical purposes. Nucleic acid testing was performed according to the Technical Guidelines for SARS-CoV-2 Laboratory Testing (from the third to fifth edition), Chinese version, issued by the National Health Commission of the People’s Republic of China.

Serum ACE2 protein was detected by enzyme-linked immunosorbent assay (ELISA) (Xiamen Lunchangshuo Biotechnology Co., LTD, China, Code No. ED-12494) in healthy subjects. The plate was pre-coated with ACE2 antibody. The standard solution was reconstituted by serial diluting with distilled water to make the standard curve. The samples and standards were added to the appropriate wells, then horseradish peroxidase (HRP) coated antibody was added. The stop solution was used to terminate the chromogenic reaction. The value was detected by the spectrophotometer. The concentration of ACE2 was calculated from the standard curve based on the absorbance values.

We used flow cytometry to determine the binding of S protein and erythrocyte. After obtaining the whole blood of healthy people, the lower hematocrit was taken after standing and stratification, washed with PBS, added with S protein (Sino Biological Inc, China, Code No.40592-V08H) and incubated at room temperature for 2 h, then added with His-tag antibody (Abcam biological, UK, Code No. ab1206) for 15 min, and CD235a (APC) antibody (Biolegend biological, USA, Code No. 349114) for 15 min before loading. Demographic data such as age, sex, and medical records including results of laboratory testing, chest radiographs, medical history, and clinical outcomes were collected from all participants. The clinical outcomes (e.g., development of ARDS or AKI, discharge, and mortality) were monitored until the last date of follow-up, March 20, 2020.

### Statistical analysis

Continuous variables were expressed as median (25th and 75th percentiles), while categorical variables were expressed as percentages. The Mann–Whitney *U*-test or the Kruskal–Wallis *H* test, as appropriate, were used to compare the medians of continuous variables between or among groups. The means of multiple groups were compared by one-way analysis of variance to detect main effect differences, and the pairwise means between groups were compared by LSD-*t*-test. Categorical data were tested using a *χ*^2^ test or Fisher’s exact test. The unadjusted associations of ABO blood type and SARS-CoV-2 infection, development of ARDS or AKI, and death were tested separately among the cohorts using Pearson’s *χ*^2^ test. Odds ratios (ORs) and 95% confidence intervals (CI) were calculated to describe the possible effect on the occurrence and development of COVID-19.

Given the limitations of a single-factor analysis, we used multivariable logistic regression to further adjust the association of ABO blood type with the occurrence of ARDS, AKI, or death for potential confounders, including the common risk factors of age, sex, pre-existing pulmonary disease, history of cardiovascular disease, diabetes mellitus, hypertension, and chronic kidney disease in the critical cohort. The history of diabetes mellitus was selected as a crucial confounder that needs to be adjusted because diabetes was reported to be protective against the development of ARDS, but as a risk factor for AKI and death (30). Most importantly, it has been associated with the ABO blood group (4). All baseline variables listed in Table 2 that had an unadjusted association with ARDS, AKI, death, or ABO blood type at *p* < 0.20 were considered for inclusion in the multivariable models (31). Additional potential confounders were only included in the final multivariable model if they altered the unadjusted OR for the association between ABO blood type and clinical outcomes by >10% in the bivariate analysis (32).

**Table 2:** Frequency and odds ratio of ABO blood types in mild and critical cohort.

Blood type	Mild cohort			Critical cohort			Chinese population (%)
	Frequency (%)	OR (95% CI)^*^	*p* value^*^	Frequency (%)	OR (95% CI)^*^	*p* value^*^	
A	35.76 (*n* = 54)^#^	1.40 (1.01, 1.96)	0.045	39.22 (*n* = 40)^§^	1.63 (1.10, 2.42)	0.015	28.39
B	22.52 (*n* = 34)	0.70 (0.48, 1.03)	0.066	29.41 (*n* = 30)	1.00 (0.66, 1.54)	0.986	29.33
O	32.45 (*n* = 49)	0.97 (0.69, 1.36)	0.845	26.47 (*n* = 27)	0.72 (0.47, 1.13)	0.149	33.20

OR, odds ratio; CI, confidence interval; *which compared with the corresponding non-A, non-B, or non-O blood type data referred to Chinese population. The frequencies for the Chinese population are estimated based on previous literature. ^#,**§**^Represents a statistically significant difference compared with the proportion of type A in Chinese population and ^#^*p* = 0.000, ^**§**^*p* = 0.005.

For statistical presentation, each blood group was compared with a reference group (blood type O or B). A stratified analysis of a reported region was not performed given that there were no substantial differences in allele distributions and divergent genetic backgrounds in participants of Han nationality between different regions. Statistical analyses were performed using SPSS (version 22.0) for Windows. *p* < 0.05 was considered as statistical significance.

## Results

### Characteristics and outcomes of the study cohorts

**Figure 1:**
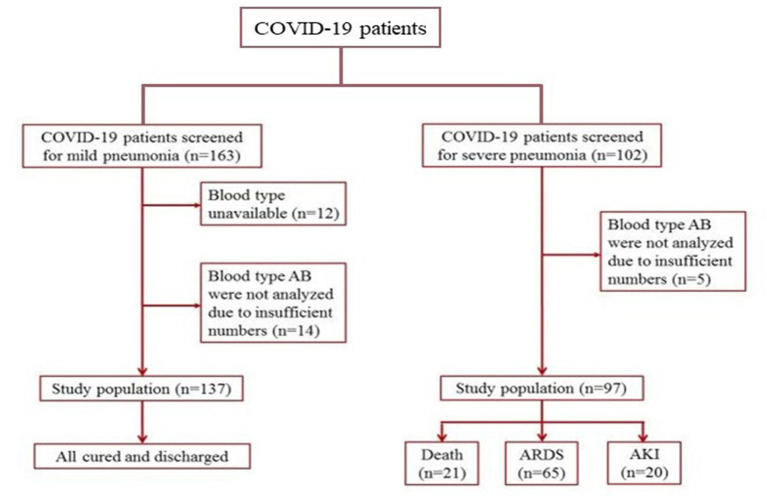
Patient inclusion and exclusion scheme. Patients with mild and severe pneumonia were enrolled in the cohorts, respectively.

In the current study, we enrolled 137 patients with mild pneumonia and 97 patients with severe pneumonia in the mild and critical cohorts, respectively (Figure 1). The demographic data, clinical characteristics, and incidence of ARDS, AKI, and death of the enrolled patients with different blood types are presented in Tables 3, 4. In this study, the individuals with AB blood type were not statistically analyzed because of the insufficient number of patients (14 in the mild cohort and 5 in the critical cohort). In addition, individuals with blood type AB only accounted for 9.0% of the Chinese population (33).

**Table 3:** Demographic data and clinical characteristics of mild cohort patients.

Characteristics	A (*n* = 54)	B (*n* = 34)	O (*n* = 49)	*p-*value	All patients (*n* = 137)
Age (years)	56 (49, 65)	50 (40, 62)	48 (36, 57)	0.187	52 (40, 64)
Age ≥ 60 years	21 (38.9%)	10 (29.4%)	19 (38.8%)	0.613	50 (36.5%)
Male	31 (57.4%)	18 (52.9%)	25 (51.0%)	0.726	74 (54.0%)
Diabetes	5 (9.3%)	4 (11.8%)	4 (8.2%)	0.857	13 (9.5%)
Hypertension	6 (11.1%)	8 (23.5%)	8 (16.3%)	0.303	22 (16.1%)
Stay in isolation wards (days)	27 (17, 35)	26 (12, 38)	22 (15.32)	0.629	25 (14, 33)

Data are shown as n (%) for categorical variables and median (interquartile range) for continuous variables.

**Table 4:** Demographic data and clinical characteristics of critical cohort patients.

Characteristics	A (*n* = 40)	B (*n* = 30)	O (*n* = 27)	*p-*value	All patients (*n* = 97)
Age (years)	68 (57, 74)	65 (58, 73)	67 (61, 75)	0.608	67 (57, 75)
Age ≥ 60 years	28 (70.0%)	22 (73.3%)	21 (77.7%)	0.780	71 (73.2%)
Male	23 (57.5%)	19 (63.3%)	17 (63.0%)	0.854	59 (60.8%)
Diabetes	10 (25.0%)	6 (20.0%)	10 (37.0%)	0.330	26 (26.8%)
Hypertension	17 (42.5%)	15 (50.0%)	15 (55.6%)	0.565	47 (48.5%)
Chronic kidney disease	2 (5.0%)	2 (6.7%)	4 (14.8%)	0.334	8 (8.2%)
Cardiovascular disease	13 (32.5%)	12 (40.0%)	5 (18.5%)	0.207	30 (30.9%)
Chronic lung disease	6 (15.0%)	1 (3.3%)	1 (3.7%)	0.128	8 (8.2%)
Clinical outcomes					
ARDS	25 (62.5%)	22 (73.3%)	18 (66.7%)	0.634	65 (67.0%)
AKI	7 (17.5%)	7 (23.3%)	6 (22.2%)	0.813	20 (20.6%)
Death	8 (20.0%)	7 (23.3%)	6 (22.2%)	0.942	21 (21.6%)

Data are shown as *n* (%) for categorical variables and median (interquartile range) for continuous variables. ARDS, acute respiratory distress syndrome; AKI, acute kidney injury.

In all 234 patients, 133 (56.8%) sufferers were men. When taking age into account, we noticed 67 (57–75) years of age in the critical cohort, and most of them (73.2%) were > 60 years of age, but 52 (40–64) and 36.5% in the mild cohort, suggesting that aged sufferers are inclined to develop into the critical ill stage.

All patients in the mild cohort recovered and were discharged. Approximately 67% of patients diagnosed with severe pneumonia developed ARDS. In critically ill patients, 21 (21.6%) died due to multiple organ failure. There was no difference in the constituent ratios of patient characteristics and outcomes among blood types A, B, and O in the two independent cohorts. Overall, the difference between baseline variables was not statistically significant in the two cohorts in terms of blood type distribution.

The demographics of healthy participants are shown in Table 1. The total number of healthy participants was 79, including 20 with type A, B, and O, and 19 with type AB. The results of statistical analysis showed no significant differences in age and gender among the healthy participants with different blood types.

### Frequencies of ABO blood in COVID-19

We then investigated the potential susceptibility of individuals with different ABO blood types to COVID-19. Table 2 shows the distribution features of the ABO blood types in the mild and critical cohorts. The proportion of blood group A in patients with COVID-19, 35.76% in the mild cohort and 39.22% in the critical cohort, was significantly higher than that in the reference population (*p* = 0.000 and *p* = 0.005, respectively), which comprised half a million Han Chinese individuals from China (33). These results imply that the blood group A population may be more vulnerable to SARS-CoV-2 infection.

In addition, blood type A was associated with an increased risk of SARS-CoV-2 infection compared with the non-A blood type, with an OR value of 1.40 (95% CI, 1.01–1.96) and 1.63 (95% CI, 1.10–2.42) in the mild and critical cohorts, respectively. However, similar results were not obtained for B and O blood types. These observations further demonstrated that the blood group A population may be more susceptible to SARS-CoV-2 infection. This is consistent with the results of the Zhao et al.’s study (34).

Compared with the frequency of O blood type in the mild cohort (32.45%) and in the Chinese population (33.20%), the distribution was lower in the critical cohort (26.47%). The OR value in the blood type O group relative to non-O blood type was not significantly decreased because of the limited number of enrolled patients (OR = 0.72, *p* = 0.149). These data suggested that less population with blood type O got critically ill.

### Association between blood type and clinical outcome

We further explored the relationship between blood type and clinical outcomes of critical patients with severe pneumonia caused by SARS-CoV-2. Demographics and clinical variables of patients with different clinical outcomes in the critical cohort are shown in Table 5. The age distribution of patients was statistically significant between the ARDS and non-ARDS, AKI and non-AKI, death, and survival groups, thus suggesting that it is closely related to the clinical outcomes and should be included in the logistic model. Although no difference was observed in the gender and the most medical histories between groups, all baseline variables presented in Table 5 were included in the multivariable logistic regression analysis, given their potential influence on patients’ clinical outcomes.

**Table 5:** Critical cohort demographics and clinical characteristics based on clinical outcomes.

Clinical variable	ARDS	Non-ARDS	*P-*value	AKI	Non-AKI	*P-*value	Death	Survival	*P-*value
Cases	65	32		20	77		21	76	
Demographic									
Age (years)	68 (63, 75)	59 (52, 72)	0.007	73 (67, 77)	65 (57, 73)	0.036	70 (65, 76)	66 (57, 75)	0.087
Age ≥ 60 years	55 (84.6%)	16 (50.0%)	0.000	19 (95.5%)	52 (67.5%)	0.012	19 (90.5%)	52 (68.4%)	0.043
Male	38 (58.5%)	21 (65.6%)	0.497	11 (55.5%)	48 (62.3%)	0.549	10 (47.6%)	49 (64.5%)	0.161
Medical history									
Diabetes	22 (33.8%)	4 (12.5%)	0.029	7 (35.0%)	19 (24.7%)	0.353	7 (33.3%)	19 (25.0%)	0.445
Hypertension	35 (53.8%)	12 (37.5%)	0.130	12 (60.0%)	35 (45.5%)	0.246	10 (47.6%)	37 (48.7%)	0.931
Chronic kidney disease	7 (10.8%)	1 (3.1%)	0.198	1 (5.0%)	7 (9.1%)	0.688	0 (0.0%)	8 (10.5%)	0.195
Cardiovascular disease	23 (35.4%)	7 (21.9%)	0.176	5 (25.0%)	25 (32.5%)	0.520	7 (33.3%)	23 (30.3%)	0.788
Chronic lung disease	5 (7.7%)	3 (9.4%)	0.777	2 (10.0%)	6 (7.8%)	0.667	3 (14.3%)	5 (6.6%)	0.365
Blood type									
A	25 (38.5%)	15 (46.9%)	0.634	7 (35.0%)	33 (42.9%)	0.813	8 (38.1%)	32 (42.1%)	0.942
B	22 (33.8%)	8 (25.0%)		7 (35.0%)	23 (29.9%)		7 (33.3%)	23 (30.3%)	
O	18 (27.7%)	9 (28.1%)		6 (30.0%)	21 (27.3%)		6 (28.6%)	21 (27.6%)	

Data are shown as *n* (%) for categorical variables and median (interquartile range) for continuous variables. ARDS, acute respiratory distress syndrome; AKI, acute kidney injury.

In the multivariable logistic regression analysis, which was used to adjust the association of ABO blood type with clinical outcomes for potential confounders, blood type A was not associated with a higher risk for ARDS, AKI, and death relative to blood type O among patients in the critical cohort (Table 6). Comparisons between blood type A and blood type B did not present any statistically significant associations in the logistic model (data not shown). These data indicate that the blood type of SARS-CoV-2-infected patients is not a potent factor that influences the development and outcome of COVID-19. When the age of patients (<60 and ≥60 years of age) was converted into an ordinal category, results showed that there was an association with ARDS (OR: 6.22 [95% CI: 2.32–16.71]) or death (OR: 9.23 [95% CI: 1.17–72.85]) when adjusted for age.

**Table 6:** Association of blood type with ARDS, AKI, and death risk in critical patients with COVID-19.

Blood type	ARDS				AKI				Death			
	Unadjusted OR (95% CI)	*P-*value	Adjusted OR (95% CI)	*P-*value	Unadjusted OR (95% CI)	*P-*value	Adjusted OR (95% CI)	*P-*value	Unadjusted OR (95% CI)	*P-*value	Adjusted OR (95% CI)	*P-value*
A	0.83 (0.30, 2.32)	0.727	1.31 (0.38, 4.53)	0.671	0.74 (0.22, 2.52)	0.632	1.19 (0.29, 4.90)	0.809	0.88 (0.27, 2.89)	0.826	0.79 (0.19, 3.20)	0.741
B	1.38 (0.44, 4.29)	0.583	2.12 (0.55, 8.15)00	0.275	1.07 (0.31, 3.68)	0.920	1.89 (0.43, 8.18)	0.398	1.07 (0.31, 3.68)	0.920	1.20 (0.28, 5.13)	0.814
O	1.00	-	1.00	-	1.00	-	1.00	-	1.00	-	1.00	-

ARDS, acute respiratory distress syndrome; AKI, acute kidney injury; CI, confidence interval; OR, odds ratio; ORs and *p-*values were determined for the comparison of blood type A or B with blood type O through logistic regression using backward elimination. Adjusted ORs for age, sex, and medical history.

### ACE2 levels and binding of S protein is different in ABO blood type individuals

**Figure 2:**
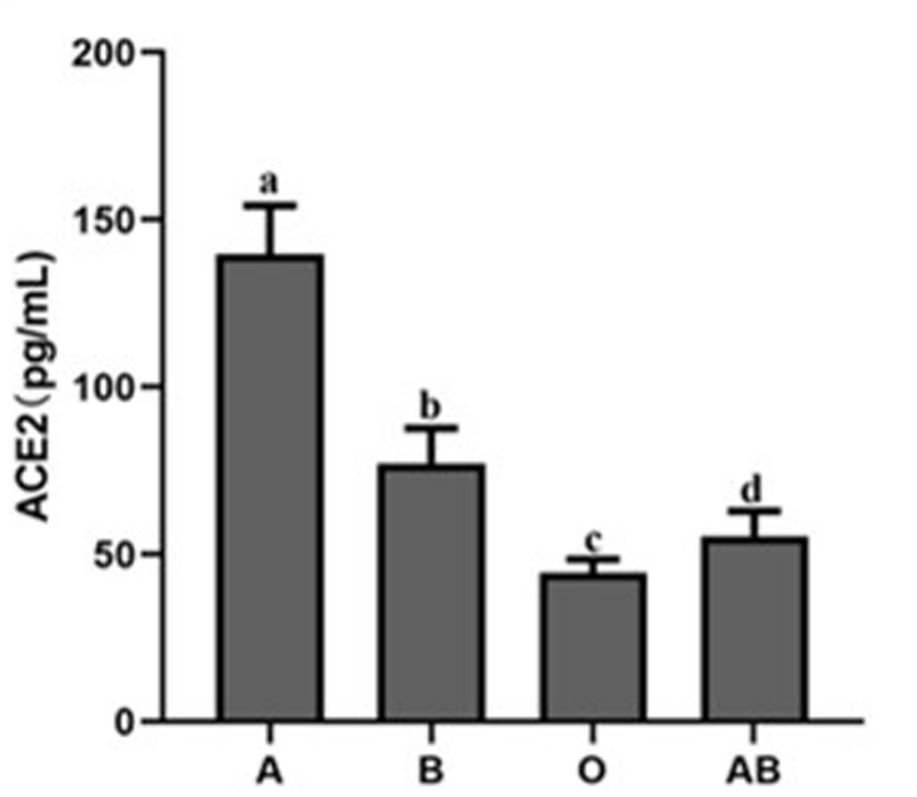
ACE2 protein expression level in the serum of healthy people with different blood groups. The different letters (a−d) on the bar chart represent statistically significant differences between groups (*p* < 0.05). ACE2, angiotensin-converting enzyme 2.

**Figure 3:**
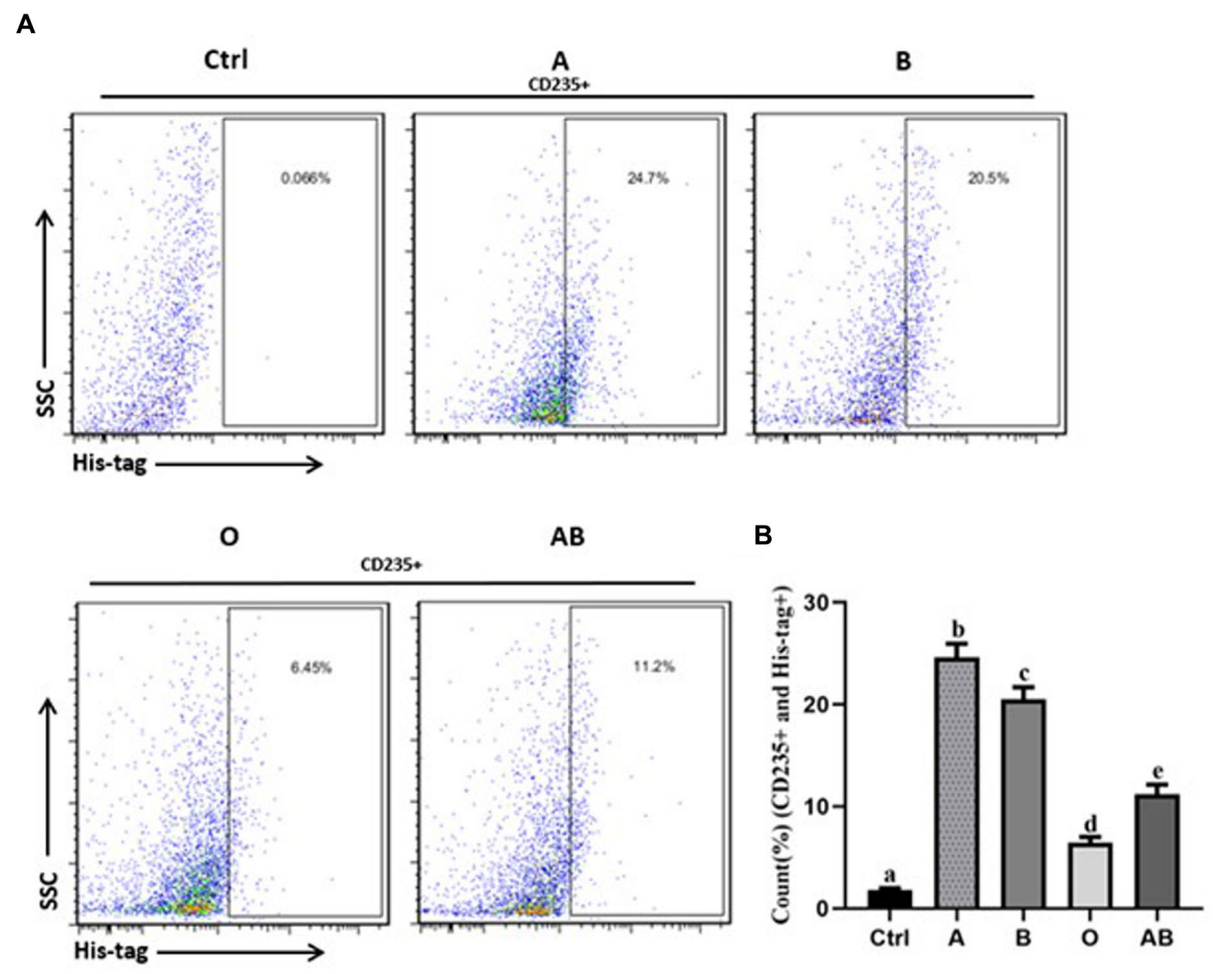
The binding rates of virus S protein to red blood cells of healthy people with different blood types. **(A)** Flow cytometry of each group. The percentage on the right represents the binding rate of S protein to red blood cells in each group (CD235+ and His-tag+). **(B)** Bar diagram of the binding rate of viral S protein to red blood cells of healthy people with different blood types. Control stands for the samples of the CD235+ red blood cells without staining with His-tag antibody. The different letters (a−d) on the bar chart represent statistically significant differences between groups (*p* < 0.05). SSC, side scatter.

To evaluate the ACE2 protein level, we collected serum from the volunteers. As shown in Figure 2, there were great differences in the ACE2 levels among different ABO blood groups. Compared to the blood type O population, the levels of ACE2 protein in the blood type A and B population were significantly higher. In addition, the ACE2 level in the blood type A population reached nearly 150 pg./ml, which was much beyond that of the B blood type population. The data indicated that individuals with blood type A have higher ACE2 level than any other blood type. Since the S protein of COVID-19 is a key factor to bind hosts, we used flow cytometry to determine the binding. As shown in Figures 3A,B, the different binding activity of S protein in ABO blood group red blood cells was obvious. Compared with type A erythrocytes, the S protein binding to type B, O, and AB erythrocytes were significantly lower, while the binding capability of blood type O erythrocytes was significantly lower than that of other groups. These data suggested that different ACE2 levels and S protein binding may be responsible for the clinical susceptibility in this study.

## Discussion

The current retrospective investigation demonstrated that blood type A individuals may be more sensitive to SARS-CoV-2. Although patient age was a factor of mortality in the present study as reported in previous studies (1), blood type distribution was not a relevant factor of ARDS, AKI, and mortality in COVID-19 patients. To our knowledge, this is the first study to report a conceivable association between ABO blood types and ARDS, AKI, and death risk, and to demonstrate that ABO glycobiology may not play a crucial role in the development of COVID-19. We believe that these findings are indicative since they probed the relationship between blood types and ARDS, AKI, and mortality, in addition to susceptibility or age-related mortality in COVID-19 sufferers.

COVID-19 now poses a great threat to human health worldwide. Thus, global efforts are being made to fight against this serious virus infection. Recently, scholars have observed that age and gender are correlated with SARS-CoV-2 infection susceptibility or severity (1). What we found in this study provided some new information from the blood type distribution angle in the mild and critical cohorts. ARDS, AKI, and mortality were not correlated with blood type distribution in COVID-19 patients. Our observations partially clarified the issues regarding blood type discrimination in the COVID-19 fighting battle. Since blood type may not be a risk factor for ARDS, AKI, and mortality, individuals with a certain blood group might not be estimated by genetic susceptibility for the poor prognosis risk. This kind of research method also may be useful for evaluating the relationship between blood type and other diseases, such as West Nile virus infection, hepatitis C, diabetes mellitus, and so on. The susceptibility of different ABO blood groups to these diseases has also been investigated, while the progression or prognosis has not been analyzed yet (4, 35, 36).

The correlation between age, gender, and susceptibility to SARS-CoV-2 has been investigated in the past several months. What we observed in the current study also confirmed that the aging population is vulnerable to SARS-CoV-2 infection. Consequently, specific preventive measures and therapies should be implemented in the aging population. In addition, the blood type A population was also more susceptible to SARS-CoV-2 infection, which was consistent with the results of previous similar investigations on West Nile virus infection and plasmodium falciparum susceptibility (35, 37). Although the sample in our study was relatively small, the data used in the study were obtained from multiple medical centers and may better represent this patient group. Further large-scale investigations are needed to resolve this concern.

It has been reported in the past that people with blood type O have a lower susceptibility to the SARS virus (38, 39). Further research found that people with blood type O had the lowest level of ACE2 protein and those with type A had the highest level. In addition, the binding rate of S protein to red blood cells was the lowest in people with type O and the highest in people with type A. We hypothesize that the main reason is the competitive inhibition of S protein binding to ACE2 by anti-A and anti-B individuals in type O populations (40). Other studies have shown that antigens A, B, and AB possess A/B phenotypic determinative enzymes, which stimulate carbohydrate aggregation, promote carbohydrate-carbohydrate interactions (CCIs), and ultimately increase the specific binding of S protein to ACE2, promoting SARS-Cov-2 infection (41). However, the specific mechanism has not been clarified, and further molecular experiments are needed to prove it. Actually, in most situations, why some diseases occur in a specific blood type population is not yet clear, except that Duffy blood type defects determine the malaria infection in Africa. Apart from the regular expression on the red blood cell surface, blood group antigens are also widely distributed on various cells and sometimes in body fluids, including respiratory epithelial cells and alveolar epithelial cells. We believe that the genetic susceptibility of blood-type glycophorin may function *via* receptor-mediated affinity binding, especially in the invasion mechanism. Blood group antigens are proven to be effective receptors for several infectious microorganisms (42).

Given the effects on inflammation, endothelial function, and microvascular coagulation of ABO glycoproteins by changing blood concentrations of soluble ICAM-1, selectins, vWF, and thrombomodulin (7, 43, 44), the ABO glycans may be important mediators of ARDS or AKI in critically ill patients. Previous studies showed that blood type A was associated with a higher risk of developing ARDS and AKI in the ICU among individuals of European descent, but not in those of African descent (7, 44). This means that racial divergence and environmental factors may alter the ABO-ARDS or ABO-AKI associations. It is plausible to hypothesize that the existing conclusion drawn in European descent with trauma or severe sepsis may not be applied to the Asian population with COVID-19. Furthermore, there is insufficient evidence regarding the pathophysiologic mechanism and features of ARDS or AKI that occurs secondary to SARS-CoV-2 infection (6, 7, 43, 44).

The present study has several limitations. First, it is not feasible to use a prospective cohort to determine when patients with mild symptoms progress to the critically ill stage since the epidemic situation was very serious at that time and all the resources were in use for emergency care. Second, there are no existing wild cohorts with available demographic and clinical characteristics that can be used as a reference group because patients who developed fever without SARS-CoV-2 infection were not identified during the corresponding period. Thus, it is impossible to analyze the correlation between blood type distribution and COVID-19 using a multiple-factor model logistic model. We had to use the chi-square test and calculate the OR value to provide an estimate. This strategy cannot rule out the possibility that blood type is the only factor associated with SARS-CoV-2 infection and progression. Our observations were only based on the results of the epidemic data analysis. The biological evidence between blood type and SARS-CoV-2 susceptibility and progression requires further cellular and molecular investigation.

The current retrospective study of two cohorts confirmed that blood type A populations are more susceptible to SARS-CoV-2 infection. However, the outcomes including ARDS, AKI, and death are poor irrespective of ABO blood group distribution in critically ill patients with COVID-19, although blood type O sufferers are unlikely to progress into the critical stage after SARS-CoV-2 infection. In addition, we further found that healthy people with blood type O had the lower ACE2 protein level and binding capability of S protein when compared other blood types. Therefore, we believe that ACE2 difference plays an important role in the SARS-CoV-2 susceptibility infection in humans. These findings are significant since they deeply probed the correlation between blood type and progression or prognosis in addition to susceptibility. It will provide general epidemic prevention guidance for the protection of the susceptible population, and further explore the infection mechanism of SARS-CoV-2. It provides a theoretical basis for the establishment of a SARS-CoV-2 susceptibility scoring system based on existing susceptibility factors and the development of gene-targeting drugs.

## Data availability statement

The original contributions presented in the study are included in the article/supplementary material, further inquiries can be directed to the corresponding authors.

## Ethics statement

The studies involving human participants were reviewed and approved by the Medical Ethics Committee of Xi’an AREA Medical Diagnostic Lab Center [Xi’an, China; approval no. XAMDL-MEC2021001]. The patients/participants provided their written informed consent to participate in this study.

## Author contributions

XZ, DL, and XH conceived and designed the study. JK, HF, FH, XM, and HL performed the experiments and contributed to the acquisition of data. HF, NL, MT, and JZ interpreted the data and contributed to statistical analysis. XZ, DL, HL, and XH drafted the manuscript. All authors read and approved the final manuscript.

## Funding

This study was financially supported by Natural Science Foundation of Shaanxi Province, China (S2021-JC-YB-0299).

## Conflict of interest

The authors declare that the research was conducted in the absence of any commercial or financial relationships that could be construed as a potential conflict of interest.

## Publisher’s note

All claims expressed in this article are solely those of the authors and do not necessarily represent those of their affiliated organizations, or those of the publisher, the editors and the reviewers. Any product that may be evaluated in this article, or claim that may be made by its manufacturer, is not guaranteed or endorsed by the publisher.
